# Risk Models to Predict Hypertension: A Systematic Review

**DOI:** 10.1371/journal.pone.0067370

**Published:** 2013-07-05

**Authors:** Justin B. Echouffo-Tcheugui, G. David Batty, Mika Kivimäki, Andre P. Kengne

**Affiliations:** 1 Hubert Department of Global Health, Rollins School of Public Health, Emory University, Atlanta, Georgia, United States of America; 2 Department of Epidemiology and Public Health, University College London, London, United Kingdom; 3 South African Medical Research Council and University of Cape Town, Cape Town, South Africa; 4 The George Institute for Global Health, Sydney, Australia; 5 Julius Center for Health Sciences and Primary Care, University Medical Center Utrecht, Utrecht, The Netherlands; Universidad Peruana de Ciencias Aplicadas (UPC), Peru

## Abstract

**Background:**

As well as being a risk factor for cardiovascular disease, hypertension is also a health condition in its own right. Risk prediction models may be of value in identifying those individuals at risk of developing hypertension who are likely to benefit most from interventions.

**Methods and Findings:**

To synthesize existing evidence on the performance of these models, we searched MEDLINE and EMBASE; examined bibliographies of retrieved articles; contacted experts in the field; and searched our own files. Dual review of identified studies was conducted. Included studies had to report on the development, validation, or impact analysis of a hypertension risk prediction model. For each publication, information was extracted on study design and characteristics, predictors, model discrimination, calibration and reclassification ability, validation and impact analysis. Eleven studies reporting on 15 different hypertension prediction risk models were identified. Age, sex, body mass index, diabetes status, and blood pressure variables were the most common predictor variables included in models. Most risk models had acceptable-to-good discriminatory ability (C-statistic>0.70) in the derivation sample. Calibration was less commonly assessed, but overall acceptable. Two hypertension risk models, the Framingham and Hopkins, have been externally validated, displaying acceptable-to-good discrimination, and C-statistic ranging from 0.71 to 0.81. Lack of individual-level data precluded analyses of the risk models in subgroups.

**Conclusions:**

The discrimination ability of existing hypertension risk prediction tools is acceptable, but the impact of using these tools on prescriptions and outcomes of hypertension prevention is unclear.

## Introduction

Hypertension has major public health and economic implications [1], [2]. Worldwide, raised blood pressure is estimated to cause around 13% deaths [3]. A growing proportion of people have prehypertension, that is, blood pressure which is higher than normal but does not meet the threshold for hypertension; in the US, for example, up to 31% of the population are so classified [4], [5]. The lifetime risk of developing hypertension may be as high as 90% [6] and over a third of adults with prehypertension progress to hypertension within a 4 year period [7].

Randomized trials of treating individuals with prehypertension suggest that hypertension onset can be prevented or delayed with drug treatment [8], or lifestyle modification [9], [10], or both. However, the most appropriate strategies to achieve effective hypertension prevention in practice are unclear. One strategy is to target individuals who are at high risk of developing hypertension. Evidence from prospective cohort studies suggests that the risk for progression to hypertension is not only determined by the status of prehypertension but depends on several factors, such as age, body mass index, blood pressure, smoking, family history, and physical inactivity [7]. Combining these known risk factors into a multivariable model for risk stratification may allow large-scale identification of the segment of the population that would benefit the most from primary prevention of hypertension. While multivariable models to predict hypertension are increasingly common, the total evidence on their performance has not been synthesized. Thus, unsurprisingly, predictive models are seldom being utilized in clinical practice in order to improve decision making and outcomes of hypertension prevention.

We report on the first systematic review of studies describing risk equations to predict hypertension. Our objective is to identify existing risk engines and, to summarize evidence as to their performance. Additionally, we will provide an overview of evidence of the impact of these hypertension risk models on decision making and the outcomes of care. Ultimately, our aim is to provide healthcare providers with a balanced account of the performance of the existing hypertension risk models.

## Methods

We conducted literature searches to identify all risk models developed to predict the future occurrence of hypertension among people with normal blood pressure or classified as prehypertension [11]. We also searched for all studies that applied existing hypertension prediction models either in the population from which the model was developed or in different populations. Lastly, we searched for reports describing impact analysis of the hypertension risk models – that is, studies examining the influence, if any, of adopting a risk model on decision making and the outcomes of care.

### Identification of Studies on Model Development and Validation

#### Data sources and searches

We utilized a four-pronged approach to identifying relevant publications. First, we searched electronic databases PubMed (Medline) and EMBASE from their inception to April 2013, for English or French language studies of development and/or validation of hypertension risk prediction models. We used a combination of search terms related to hypertension and prediction. The search strategies are provided in detail in Text S1, and the last search date was April 30, 2013. Second, we manually searched the reference sections of retrieved publications, and identified any citations through the ISI Web of Science for additional published and unpublished data. Third, we contacted experts in the field, and finally, we searched our own files.

#### Study selection

**Figure 1:**
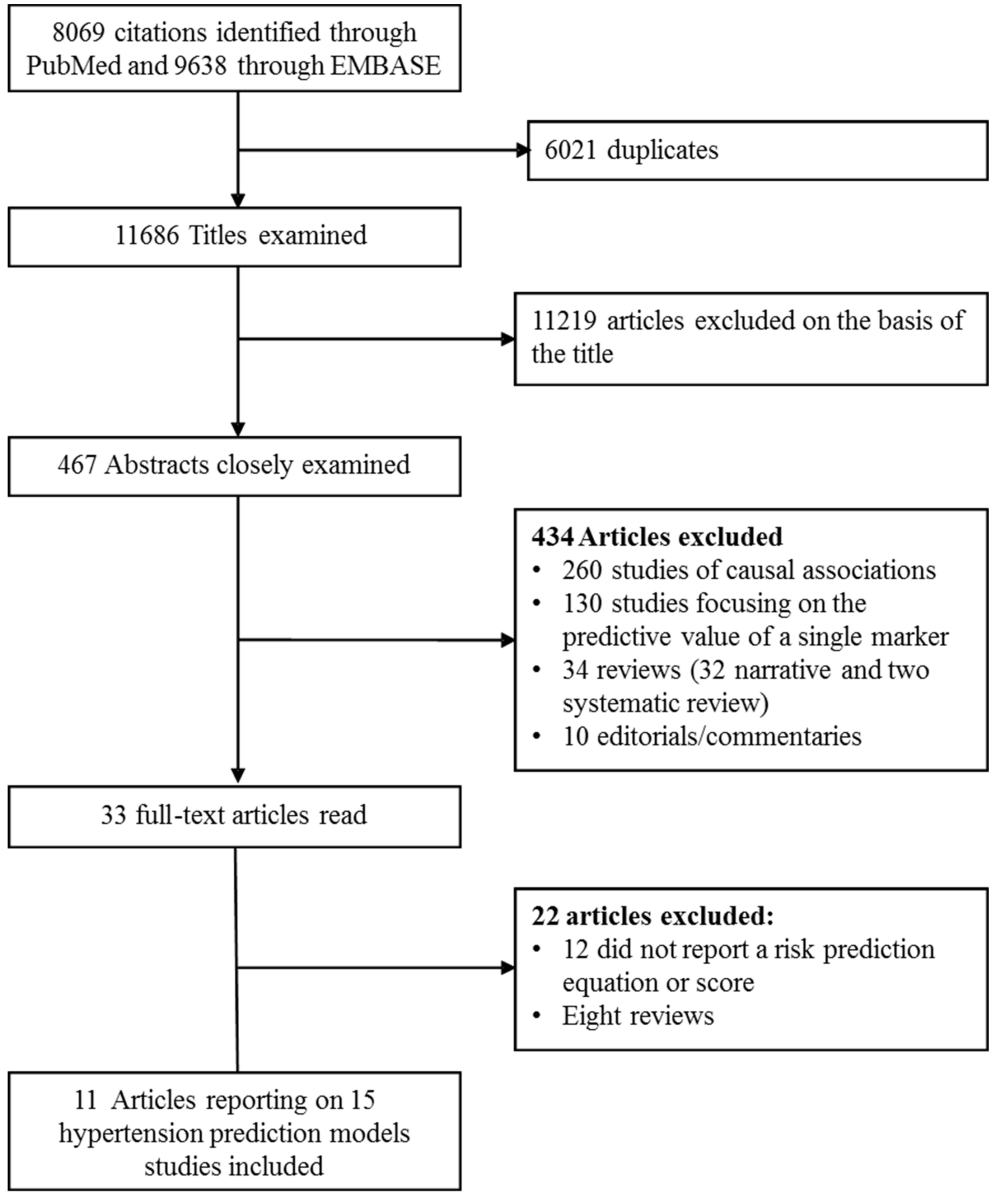
Identification of relevant publications.

Two experienced evaluators (JBE, APK) independently identified articles and sequentially screened them for inclusion (Figure 1). Where necessary, the full text of articles and/or supplemental materials (tables and appendices) was reviewed before deciding on the inclusion. Disagreements were solved by a third investigator (GDB).

Eligible articles had to report on a risk assessment tool (equation and/or score) for predicting hypertension occurrence, and be based on adult human populations. Reporting of quantitative measures of the performance of models was preferable but not necessary for inclusion. We excluded studies that only reported measures of association between risk factors and incident hypertension, and simulation studies. We also excluded studies of prediction of gestation-related hypertensive disorders (e.g., preeclampsia).

#### Data extraction and quality assessment

Two experienced reviewers (JBE and APK) independently conducted the data extraction and quality assessment. We did not use a particular framework for quality assessment, as there is no consensus over a quality assessment framework for risk prediction models [12]. From each study, we extracted data on study design, setting, population characteristics, number of patients in the derivation and validation cohorts, the number of participants with the outcome of interest, the number of candidate variables tested as predictor and the numbers and list of those included in the final model, as well as the type of statistical model used. For the discriminative ability of models, we extracted information on the area under the receiver operating characteristic curve (AUC) or C-statistic. To describe model calibration, we extracted data on the difference between the observed and predicted rates of hypertension, as well as the p-value of the corresponding test statistic. For the assessment of reclassification, we extracted the net reclassification improvement (NRI) and integrated discrimination improvement index (IDI) values, as well as their respective 95% CIs and p-values, when available [13], [14].

#### Data synthesis and analysis

The wide range of metrics used for the assessment of the predictive ability of hypertension risk models, and the heterogeneity in both the risk factors used for prediction and their number, as well as the study designs precluded any reliable data synthesis in the form of meta-analysis. We therefore opted to conduct a narrative synthesis of the evidence. However, as a number of studies evaluated the Framingham hypertension risk model [15], thus representing a subgroup of less heterogeneous studies, we applied the random-effects model meta-analyses to combine the estimates of the AUC from those studies and assessed the between-study heterogeneity, with the use of the R statistical software version 2.13.0 [13-04-2011], (The R Foundation for Statistical Computing, Vienna, Austria).

### Impact Analysis of Hypertension Risk Models

Impact analysis studies were captured by: 1) scanning the publications identified through the search strategy for model development and validation, and 2) applying the search strategy for impact studies proposed by Reilly and Evans [16], which combines the model’s acronym or name of the cohort or first author, with specific search term combination (Text S2).

## Results

The study selection process is described in Figure 1. After scanning titles of the citations identified through searches, 467 abstracts were selected for in-depth evaluation and 33 full-text publications were reviewed. After all exclusions, eleven articles reporting on 15 different hypertension risk prediction models met the eligibility criteria and were included in the review.

Table 1 summarizes the characteristics of studies from 11 publications that report on the development of hypertension risk prediction models. All the models were developed using cohort studies with incident hypertension as the outcome of interest.

**Table 1:** Development of risk prediction tools for predicting hypertension.

Author, Reference	Name of the risk model	Country/Ethnicity	Study design	Candidate variables (n)	Risk factors included	n outcomes/n total	Age-years	Definition of outcomes predicted	Time-horizon (years)	Discrimination(C-statistic)	Calibration	Method of validation	Type of regressionmodel
Pearson et al, 1990 [17]	Johns Hopkins	USA/Mixed, mainly White	Prospective cohort of men only	NR	Age, SBP at baseline, paternal history of hypertension, and BMI	104/1,130	25 or less	Self-reported use of BP lowering medications	30	NR	NR	NR	Cox
Parikh et al,2008 [15]	Framinghma score	USA- Mainly Whites	Prospective cohort	11	Age, sex, SBP, DBP, BMI parental hypertension (using three categories : neither, one parent, or both parents), and cigarette smoking	796/1,717	20 to 69 (54% women),	SBP≥140 mm Hg or DBP≥90 mm Hg or use of BP lowering medications	3.8	0.788	HL χ^2^ = 4.35 (*P = *0.88)	Apparent	Weibull
										NR	Overoptimism factor 0.03	Boostrapping	
Paynter et al, 2009 [18]	Women’s Health Study (WHS) Inclusive model	USA -Whites and blacks	Prospective cohort of women only	14	age, ethnicity, BMI, total grain intake, SBP, DBP, apolipoprotein B, lipoprotein (a), and C-reactive protein	1,935/9,427	45 to 64	Self-report or SBP≥140 mm Hg or DBP≥90 mm Hg	8	0.714	HL χ^2^ = 2.9 (P = 0.94)	Apparent	Logistic
										0.705	HL χ^2^ = 24.6 (P = 0.002)	Split-sample (67% vs. 33%)	
Paynter et al, 2009 [18]	Women’s Health Study (WHS) simplified model with lipids	USA -Whites and blacks	Prospective cohort women only	23	Age, BMI, SBP, DBP, ethnicity (being of Black or Hispanic race) and total to HDL- cholesterol ratio	1,935/9,427	45 to 64	Self-report or SBP≥140 mm Hg or DBP≥90 mm Hg	8	0.708	HL χ^2^ = 9.4 (P = 0.31)	Apparent	Logistic
										0.703	HL χ^2^ = 20.7 (P = 0.008)	Split-sample (67% vs. 33%)	
Paynter et al, 2009 [18]	Women’s Health Study (WHS)-simplified model	USA- Whites	Prospective Cohort women only	23	Age, BMI, race/ethnicity, SBP, and DBP	1,935/9,427	45 to 64	Self-report or SBP≥140 mm Hg or DBP≥90 mm Hg	8	0.707	HL χ^2^ = 6.0 (P = 0.64)	Apparent	Logistic
										0.703	HL χ^2^ = 12.3 (P = 0.14)	Split sample (67% vs. 33%)	
Kivimäki, et al, 2009 [20]	Whitehall II risk score	England – mainly Whites	Prospective cohort	NR	Age, sex, BMI, SBP, DBP, BMI, parental hypertension (using two categories : yes versus no), and cigarette smoking	1,258/8,207	35 to 68 (31% women)	SBP≥140 mm Hg or DBP≥90 mm Hg or use of BP lowering medications	5	NR	NR	Apparent	Weibull
										0.804	HL χ^2^ = 14.3	Split-sample (60% vs. 40%)	
Kivimäki, et al, 2010 [21]	Whitehall II Repeat measures risk score	England- mainly Whites	Prospectivecohort	NR	Age, sex, BMI, parental hypertension, current cigarette smoking, current SBP, current DBP, previous SBP, and previous DBP, age – DBP interaction.	614/4,135 for the derivation cohort and 438/2,785 for the validation cohort	35 to 68	SBP≥140 mm Hg or DBP≥90 mm Hg or use of BP lowering medications	5	NR	NR	Apparent	Weibull c
										0.799	HL χ^2^ = 6.5	Split-sample (60% vs. 40%)	
Kivimäki, et al, 2010 [21]	Whitehall II Average blood pressure measure risk scores	England- mainly Whites	Prospectivecohort	NR	Age, sex, BMI, parental hypertension, current smoking and average SBP* (current and previous SBP), average DBP(current and previous DBP), age - usual DBP interaction	614/4,135 for the derivation cohort and 438/2,785 for the validation cohort	35 to 68	SBP≥140 mm Hg or DBP≥90 mm Hg or use of BP lowering medications	5	NR	NR	Apparent	Weibull
										0.794	NR	Split-sample (60% vs.40%)	
Kivimäki, et al, 2010 [21]	Whitehall II usual measure risk scores	England- mainly Whites	Prospectivecohort	NR	Age, sex, BMI, parental hypertension, current cigarette smoking, current SBP, current DBP, usual SBP, and usual DBP **, age – current DBP interaction	614/4,135 for the derivation cohort and 438/2,785 for the validation cohort	35 to 68	SBP≥140 mm Hg or DBP≥90 mm Hg or use of BP lowering medications	NA	NR	NR	Apparent	Weibull
										0.799	NR	Split-sample (60% vs.40%)	
Kshirsagar et al, 2010 [19]	ARIC/CHS Score	USA – Mixed but mainly Whites	Prospective cohort	11	Age, SBP or DBP, smoking, family history of hypertension, diabetes mellitus, BMI, the age–DBP interaction, female sex, and lack of exercise	3,795/11,407 (7,610 for derivation sample and 3,692 for the validation sample)	45 to 64	SBP≥140 mm Hg or DBP≥90 mm Hg or reported use of BP lowering medications	3, 6, and 9	0.739 (3years), 0.755 (6 years), 0.800 (9 years) and 0.782 (ever)	NR	Apparent	Logistic
										0.751 (3 years), 0.743 (6 years), 0.773 (9 years) and 0.761 (ever)	NR	Split-sample (60% vs.40%)	
Bozorgmanesh et al, 2011 [23]	Iran BP risk score	Iran- Asians	Prospective cohort	NR	Among women: family history of premature CVD, WC, SBP, and DBP/Among men: smoking, SBP, and DBP.	805/4,656 (2,695 women)	Mean :42	SBP≥140 mm Hg or DBP≥90 mm Hg or self-reported use of BP lowering medications	6,	0.73 in women and 0.74 in men.	women (HL–χ^2^ = 7.8, P = 0.554) and men (HL χ^2^ = 8.8, P = 0.452).	Apparent	Weilbull
Chien et al, 2011 [24]	Taiwan BP clinical risk model	Taiwan -Chinese	Prospective cohort	NR	age, sex, BMI, SBP and DBP	2506/1029	≥35	SBP≥140 mmHg or DBP≥90 mmHg, and use of BP lowering medications	6.15	0.732	HL χ^2^ = 10.9 (P = 0.21)	Apparent	Weilbullc
Chien et al, 2011 [24]	Taiwan BP biochemical risk model	Taiwan- Chinese	Prospective Cohort	NR	age, sex, BMI, SBP, DBP, white blood count, fasting glucose and, uric acid	1,029/2,506	≥35	SBP≥140 mmHg or DBP≥90 mmHg, and use of BP lowering medications	6.15	0.737	HL χ^2^ = 6.4 (P = 0.60)	Apparent	Weillbull
Lim et al, 2013 [25]	Korean risk model	Korea -Asians	Prospective cohort	NR	age, sex, smoking, SBP, DBP, parental hypertension, BMI	819/4747	40–69	SBP≥140 mmHg or DBP≥90 mmHg, and use of BP lowering medications	4	0.7805	HL χ^2^ = 4.17 (P = 0.8415)	Apparent	Weillbull
										0.791		Split-sample (60% vs. 40%)	
Fava et al, 2013 [22]	Swedish risk model	Sweden –Whites	Prospective cohort	NR	age, sex, age^2^, sex times age, heart rate, obesity (BMI>30 kg/m^2^), diabetes, hypertriglyceridemia, prehypertension, family history of hypertension, sedentary in spare time, problematic alcohol behavior, married or living as a couple, high level non-manual work, smoking	NR/10 781	NR	SBP≥140 mmHg or DBP≥90 mmHg, and use of BP lowering medications	23	0.662	NR	NR	Logistic

ARIC: Atherosclerosis Risk in Communities, BMI: body mass index; BP: Blood pressure, CHS: Cardiovascular Health Study, CVD: cardiovascular disease, DBP: diastolic blood pressure, DM; diabetes mellitus, eGFR: estimated glomerular filtration rate, HF: heart failure, HDL-cholesterol: High Density Lipoprotein -Cholesterol, IDI: Integrative Discriminative Index, HTN: hypertension, Hx: history, NA: Not applicable, NRI: Net reclassification Index, NR: not reported,, SBP: systolic blood pressure, SD: standard deviation, WC: waist circumference, WHR: waist to hip ratio.

* average of the current and previous blood pressure measurements from different time points and entered this, instead of current and previous blood pressure measurements, in the risk prediction score.

** Usual systolic and diastolic blood pressures at the previous time point according to the following formula: UBPi = BPbm+ [RDR× (BPbi-BPbm)], where UBPi refers to each participant’s usual blood pressure, BPbm to the average blood pressure in the population, RDR to the regression: dilution ratio, and BPbi to the participant’s blood pressure. The regression: dilution ratio for a non-hypertensive population by using the mean values of the previous and current blood pressures, which were computed within quartiles of the previous blood pressure. The difference in mean blood pressure between the lowest and highest quartiles for the previous blood pressure and the current blood pressures were calculated and their ratio used to estimate the regression: dilution ratio.

### Populations, Outcomes and Risk Factors

Ten of the 13 hypertension risk algorithms were developed from samples drawn from populations in the USA (n = 6) [15], [17], [18], [19], UK (n = 4) [20], [21], or Sweden (n = 1) [22]; study participants mostly seemed to comprise Whites. One study included only Iranian [23], one only Chinese participants [24], and another only Koreans [25]. The number of people included in the studies ranged from 1,103 to 11,407 and the age of participants at baseline ranged from less than 25 to 69 years or more. The study population for three models comprised only women [18], and for one model only men [17]. The length of follow-up in the studies ranged from 4 to 30 years.

Except for one study [17], outcomes were defined using the Joint National Committee (JNC) - VII definition of hypertension (i.e., systolic/diastolic blood pressure > = 140/90 mmHg or use of blood pressure lowering medications) [11]. Three studies clearly provided data on the numbers of candidate variables tested for inclusion in the models [15], [18], [19]. This number ranged from 11 to 23 [15], [18], giving estimates of the number of incident hypertension cases per candidate variable ranging from 72 to 345 [15], [18]. The predictors most commonly included in the final prediction models were: age, sex, body mass index, systolic blood pressure, and diastolic blood pressure, parental history of hypertension, and cigarette smoking (Table S1). Additionally, a variable for the interaction term between age and blood pressure variable was included in four models [19], [21]. Biomarkers, such as C-reactive protein (CRP) [18], apolipoprotein A [18], and uric acid [24] were included in one model.

One model was derived using Cox regression [17], five models using logistic regressions [18], [19], [22] and the rest were developed using Weibull regression models [20], [21], [23], [24], [25]. All studies reported the original model with beta coefficients and 4 studies presented additional point-based scoring systems or charts [15], [23], [24], [25].

### Performance of Risk Prediction Models

Table 1 shows the performance of the various hypertension risk models. All the included studies reported a C-statistic ranging from 0.70 [18] to 0.80 [20], indicating an acceptable-to-good discriminatory capability. Ten scores were internally validated through split-sample validation (nine models) or bootstrapping (one model). Ten risk models had an estimate of calibration, the Hosmer-Lemeshow test statistic and accompanying p-values, which generally indicated good calibration [15], [18], [20], [21], [23], [24], [25].

### Validation of Hypertension Risk Prediction Models

Table 2 shows the results of the external validation of hypertension risk models. Two of the risk models (Framingham and Johns Hopkins) were externally validated. The Framingham hypertension risk model was validated in four independent populations [20], [24], [25], [26], while the Hopkins model was validated in one [24]. The C-statistic in validation studies (0.71 to 0.81) was generally lower than that in the derivation sample, but always acceptable. The change from the original C-statistic when the model was first derived ranged from −0.08 to +0.01 (Table 2), being negative or null except in one case where it was positive [20], thus indicating a generally lower discrimination in validation populations.

**Table 2:** External validation of risk prediction tools for hypertension.

Author, Reference	Name of the score validated	Validation population/Country	Ethnicity	Design	Sample Size (n outcomes/n total)	Age (years)	Time-horizon (years)	Discrimination AUC	Change from the original AUC when model first developed	Calibration	Reclassification	
											NRI, % (95% CI/p-value)	IDI % (95% CI/p-value)
Kivimäki, et al, 2009 [20]	Framingham score	Whitehall II Study/England	Mixed, mainly Europid	Prospective cohort	785/5,472	35 to 68	5	0.803	+0.02	HL χ^2^ = 11.5	NA	NA
Munter et al, 2010 [26]	Framingham score	MESA cohort/USA	Mixed (45%, 20%, 22%, and 13% were Whites, African-Americans, Hispanic, and Asian)	Prospective cohort	849/3,013	45 to 84	4.8	0.788	0	HL χ^2^ for predicted vs. observed (*P*<0.001); recalibrated and best-fit models fit (*P* = 0.064 and 0.245, respectively)	NR	10 (95% CI: −1.7 to 22.7) for comparing the Framingham score vs. SBP alone, and 146.0 (116.0 to 181.0) for Framingham score vs. age-specific categories DBP
Chien et al, 2011 [24]	Framingham score	Chinese/Taiwan	Asian	Prospective cohort	1,029/2,506	≥35	6.15	0.709	-0.08	HL χ^2^ = 7.4 (P = 0.49)	NA	NA
Chien et al, 2011 [24]	Hopkins score	Chinese/Taiwan	Asian	Prospective cohort	1,029/2,506	≥35	6.15	0.707	−0.08	HL χ^2^ = 16.7 (P = 0.03)	NA	NA
Lim et al, 2013 [25]	Framingham score	Korean Genome and Epidemiology Study/Korea	Asian	Prospective cohort	819/4747	40 to 69	4	0.789	+0.01	HL χ^2^ = 29.73, (P = 0.0002)	NA	NA

AUC, area under the receiver operating characteristic curve; CI; confidence interval; DBP: diastolic blood pressure; HL: Hosmer-Lemeshow; IDI: Integrative Discriminative Index; NA: not applicable; NR: Not reported; NRI: Net Reclassification Index; SBP, systolic blood pressure.

In a random-effects meta-analysis (Figure S1), pooled AUC for prediction of hypertension risk using the Framingham risk equation was 0.78 [95% confidence interval (CI): 0.75 to 0.81] in the four cohorts that explored the performance of this risk equation although significant heterogeneity (I^2^ = 92.5%) was evident (p<0.001).

### Model Improvements and Head-to-head Comparisons

Three studies examined the impact on the predictive capacity of the hypertension model by adding additional variables [21], [22], [24]. The Whitehall II study assessed whether prediction with the Framingham risk model was significantly improved after reclassification on the basis of new scores [21], including repeat measurement of variables in the model (NRI 9.3% (95% CI: 4.2 to 14.4), utilizing an average of blood pressure measurements (NRI: 5.8% [95% CI: 0.1 to 11.4]), and the value of usual plus current blood pressure values (NRI: 5.8% [95% CI: 0.1 to 11.4]). The findings indicated modest or no significant improvement in predictive performance. The Hopkins score investigators reported a significant improvement after adding biochemical factors (glucose level, white blood cell count, uric acid) to traditional hypertension risk factors; the difference in AUC was 0.005 (p = 0.17), NRI was 7.0% (95% CI: 3.7 to 10.3, p = 0.0002) and IDI was 1.0 (95% CI: 0.7 to 1.3, p<0.0001) [24]. The performance of a non-genetic Swedish hypertension risk model was not improved by the addition of a genetic risk score variable based on 29 independent single nucleotide polymorphisms. The AUC for the non-genetic variables-based model was 0.662 (95% CI: 0.651–0.672) and increased to 0.664 (95% CI: 0.653–0.675) with inclusion of genetic variables [22].

### Impact Analysis of Hypertension Prediction Models

We found no studies that have assessed the impact of adopting hypertension risk scores on the processes of care and outcomes of care for people without hypertension.

## Discussion

This systematic review shows the feasibility of assessing individual’s risk of acquiring a diagnosis of hypertension among people with high or normal blood pressure using a combination of commonly assessed variables. By representing a primordial approach, these multifactorial risk models for predicting hypertension occurrence add to traditional cardiovascular prediction that have been focused on disease endpoints rather than risk factors. However, most existing hypertension risk models are still at the early stages of the development and evaluation process, and only two of them have been tested in populations different from those used to develop the models.

### Strengths of Existing Models and their Utility for Patient Care

The variables used in prediction models were largely the same across various age groups, and are generally assessable in routine practice. The discriminative ability of existing models was generally acceptable-to-good in both the derivation and validation samples. With one exception [26], the included studies demonstrated that extra predictive information is gained when other variables are factored into a risk model in addition to an individual’s current blood pressure levels for predicting the probability of acquiring a diagnosis of hypertension. Furthermore, hypertension per se antedates many cardiovascular diseases such as heart failure [27] and stroke, and is associated with a shorter life expectancy [28]. Thus, prevention of new-onset hypertension may prevent the emergence of a risk factor for hard outcomes, representing primordial rather than primary or secondary prevention. Models for predicting hypertension occurrence therefore have potential public health and clinical applications in the prevention hypertension.

### Opportunities for Improving the Uptake of Hypertension Prediction Models in Practice

Practice guidelines currently recommend the use of multivariable risk models as the appropriate basis for cardiovascular diseases (CVD) risk stratification and prescription of risk reducing therapies in routine care [29], [30]. The most recommended prediction algorithms for this purpose are CVD models which incorporate data on routinely measured conventional risk factors [31], [32] which are also common to existing hypertension prediction models. There is therefore an opportunity at no extra cost for harnessing CVD and hypertension risk predictions in routine care.

Using hypertension prediction models in routine care has several potential advantages, including tailoring the prescription and intensity of preventive solutions in those at high risk of progression to hypertension, and improving shared decision making through accurate risk communication to people at high risk, with potential positive impact on adherence to prescribed interventions. Besides routine clinical settings, hypertension risk scores can also be used 1) to select people at high risk for inclusion in clinical trials of hypertension prevention; 2) to project the future burden of hypertension at a population level, and 3) to allocate resources based on mean levels of the various components of the hypertension risk score in the communities. For all these applications, estimates of hypertension risk from predictions models must be accurate and valid.

### Limitations of Existing Models and Perspectives for Future Research

None of the existing hypertension models was developed using data specifically collected for risk modelling purpose; thus raising concerns about the completeness and measurement precision of the predictors and outcomes included in the models [33]. Other potential drawbacks that may have affected model performance included dichotomisation of continuous variables prior to modelling, linearity assumption without formal testing and exclusion of participants with missing values on predictor/outcome variables.

One model was published without indicators of performance during the derivation process [17]. For models that provided measures of performance, these were mostly based on the direct application of the model on the derivation sample (apparent performance), or performance measures from internal validation (split-sample or bootstrap). The apparent performance may be overoptimistic, and thus internal validation only provides new users with a rough idea about what to expect when applying the model to their own populations. Calibration was less commonly assessed or reported during the derivation process, although it is commonly agreed that calibration is largely affected by the background risk which varies across populations. Consequently models need updating through recalibration procedures to provide accurate estimate of the risk in new populations. There have been attempts to recalibrate the Framingham hypertension risk score in new populations [20].

External validation of a model in new populations is a key step before it can be recommended for extensive use. Only two of the 13 hypertension prediction models have been tested on different populations [15], [17], and only a few times [20], [24], [26]. With regard to the most tested model, the Framingham hypertension risk model [15], our meta-analysis suggests that the model would perform equally well among whites in different settings [20], [26] but not necessarily in other ethnic groups [24]. Hence, more validation studies of existing models are needed, ideally by different group of investigators to guarantee their generalizability to a larger number of people.

In addition to providing mathematical equations, some investigators provided point-scoring systems [15], [23], [24]. While the performance of the point-scoring format of risk estimation may be lower than that of the original model, such a presentation of risk might facilitate the use of these tools among health care providers who may not be familiar with complex mathematical formulas, and consequently improve the uptake of the risk prediction tools in various settings [34]. Some context- specific efforts may also be required to derive the appropriate cut-off for defining high-risk status when those models are integrated in guidelines for screening. However, prior confirmation that the implementation of hypertension risk prediction models will affect the behavior of healthcare providers and improve outcomes of care is necessary. At present, no implementation trial of hypertension risk prediction models has been conducted.

One model relied on self-reported blood pressure/hypertension status solely to define the incidence of the outcome [18]; replication with measured blood pressure is needed to test the validity of these findings. Two studies indicated the change in the method used to measure blood pressure over the course of the cohort study [20], [21]; this was probably also the case in the other cohorts. Such a change in the measurement method may have affected the incidence of hypertension, and thus the performance of the risk score. Some of the risk score excluded people with diabetes [15], [20], thus these may not be applicable to that segment of the population. In most studies, diagnostic of high BP was based on a single visit rather than repeated visits, however one study that incorporated previous blood pressure records or estimates of average or usual blood pressure in in risk prediction models indicated that these may not improve the prediction of future hypertension [21].

Participants to the reviewed studies comprised mostly whites. A homogenous population does not allow assessment of the full extent of the variability in hypertension risk. This is a drawback given that racial/ethnic groups are particularly prone to hypertension (e.g., people of African descent). Future studies should therefore incorporate more subjects of different ethnic background.

Few studies examined the incremental predictive value of novel circulating or genetic markers of future hypertension in existing models. Matrix remodeling biomarkers (inhibitor of metalloproteinase-1 [TIMP-1] and metalloproteinase-9 [MMP-9]) [35], inflammatory markers (C-reactive protein and plasminogen activator inhibitor-1) [36], urinary albumin/creatinine ratio [36], and several genetic markers [37] have been shown to be associated with hypertension, and may be useful for hypertension risk stratification. However, some of these factors may not always be readily assessable or available in all settings, or might require more complex judgment and interpretation.

### Strengths and Limitations of the Review

The strengths of this review include the exclusion of studies that only reported effect estimates for independent association of risk factors with hypertension. Hitherto, the performance of existing hypertension risk models in terms of discrimination, calibration, and reclassification had not been critically examined. The current review has summarized those performance measures, and identified the gaps in the evidence on the prognostic ability of the existing risk prediction models. This will form the basis for future improvement. Our study is very informative as to whether or not to incorporate risk models for predicting hypertension in guidelines for evaluation and management of elevated blood pressure. Evidence for such an inclusion would be reinforced by additional studies of external validation of risk scores, as well as studies of the impact of their use on outcomes. The main limitation of this review is the lack of individual-level data that could have allowed a pooled analysis of the performance of models and subgroup analyses. Our ability to assess publication bias was also limited. Direct comparison of risk models was also limited by the lack of some relevant information in some of the published development or validation studies. Standardized reporting of the results of risk models will help avoid mistakes such as inadequate description of the performance of models, or not presenting the results in a way that can be used by clinicians. Better reporting of development and validation studies is needed to help clinicians and other decision makers identify relevant models with potential clinical value. Furthermore, the use of standard terms and nomenclature in studies of risk prediction models will allow the different laboratory, clinic, and population disciplines to interact, and for findings to be interpreted appropriately and consistently by clinicians, patients and public health practitioners.

### Conclusions

Current multifactorial risk models for predicting hypertension occurrence have an acceptable-to-good discriminative ability. However, before these tools are incorporated in guidelines, their calibration and external validation as well as incremental predictive power beyond the prehypertension status alone. Future randomized controlled trials are needed to determine the impact of adopting risk models on outcomes of care. Cost-effectiveness analyses of the application of this primordial prevention strategy would be crucial in future assessments of hypertension prediction models.

## Supporting Information

Figure S1 Summary estimate of AUCs (95% confidence interval) for hypertension risk prediction for the Framingham risk score in various validation studies. AUC, area under the receiver operating characteristic curve; CI, confidence interval.
(TIF)

Table S1 Factors included in models for predicting hypertension.
(DOCX)

Table S2 Reporting and management of missing values in studies on the development of hypertension risk scores.
(DOCX)

Text S1 Search terms for risk model development or validation studies.
(DOCX)

Text S2 Search terms for risk model impact studies.
(DOCX)

Text S3 PRISMA checklist.
(DOC)
