# Supplementary figures and images for: Risk Models to Predict Hypertension: A Systematic Review
Source: PLoS One. 2013 Jul 5;8(7):e67370. doi: 10.1371/journal.pone.0067370 (PMC3702558; doi:10.1371/journal.pone.0067370)

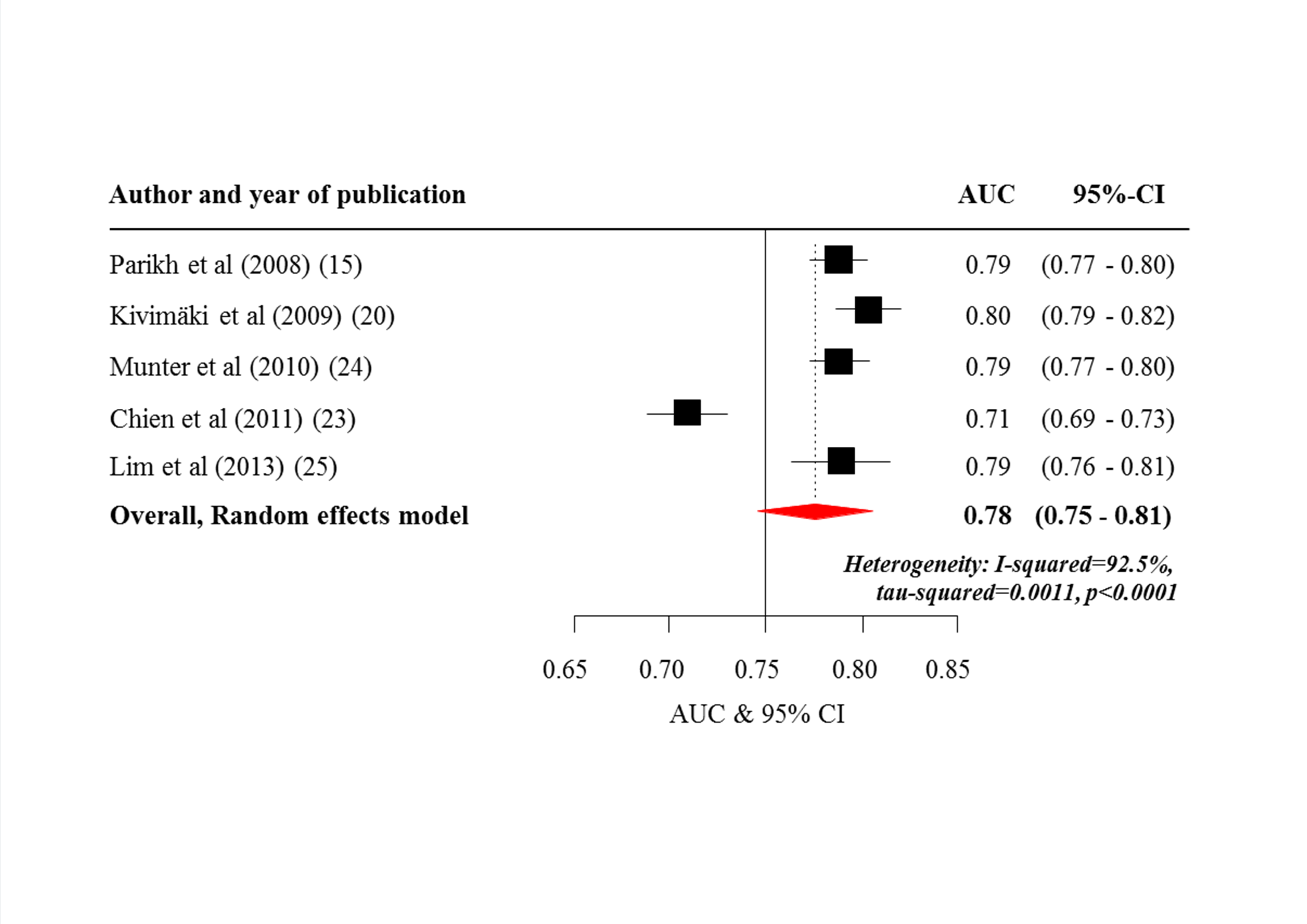

Supplement: Figure S1 — Summary estimate of AUCs (95% confidence interval) for hypertension risk prediction for the Framingham risk score in various validation studies. AUC, area under the receiver operating characteristic curve; CI, confidence interval. (TIF) [file pone.0067370.s001.tif]
