# Supplementary material for: Risk Models to Predict Hypertension: A Systematic Review
Source: PLoS One. 2013 Jul 5;8(7):e67370. doi: 10.1371/journal.pone.0067370 (PMC3702558; doi:10.1371/journal.pone.0067370)
Supplement: Table S1 — Factors included in models for predicting hypertension. (DOCX) [file pone.0067370.s002.docx]

**Table S1: Factors included in risk models tools for predicting hypertension**

| **Author, Year, Reference** | **Name of the score** | **Age** | **Gender** | **Ethnicity** | **SBP** | **DBP** | **Previous**  **BP** | **Parental HTN** | **BMI or WC** | **Smoking** | **Lipids variables** | **Physical activity** | **Dietary factors** | **Age*BP** | **Age*sex** | **Glucose levels** | **CRP** | **Uric acid** | **WBC count** | **Socio-economic variables** |
| --- | --- | --- | --- | --- | --- | --- | --- | --- | --- | --- | --- | --- | --- | --- | --- | --- | --- | --- | --- | --- |
| Pearson et al, 1990 [17] | Johns Hopkins model | Yes | No | No | Yes | No | No | Yes | Yes | No | No | No | No | No | No | No | No | No | No | No |
| Parikh et al,2008 [[15](#_ENREF_15)] | Framingham model | Yes | Yes | No | Yes | Yes | No | Yes | Yes | Yes | No | No | No | No | No | No | No | No | No | No |
| Paynter et al, 2009 [18] | WHS Inclusive model | Yes | No | Yes | Yes | Yes | No | No | Yes | No | Yes | No | Yes | No | No | No | Yes | No | No | No |
| Paynter et al, 2009 [18] | WHS simplified model with lipids | Yes | No | Yes | Yes | Yes | No | No | Yes | No | Yes | No | No | No | No | No | No | No | No | No |
| Paynter et al, 2009 [18] | WHS simplified model | Yes | No | Yes | Yes | Yes | No | No | Yes | No | No | No | No | No | No | No | No | No | No | No |
| Kivimäki, et al, 2009 [20] | Whitehall II model | No | No | No | Yes | Yes | No | No | Yes | Yes | No | No | No | No | No | No | No | No | No | No |
| Kivimäki, et al, 2010 [21] | Whitehall II repeated measure model | No | No | No | Yes | Yes | Yes | Yes | Yes | Yes | No | No | No | Yes | No | No | No | No | No | No |
| Kivimäki, et al, 2010 [21] | Whitehall II average measure model | No | No | No | Yes | Yes | Yes | Yes | Yes | Yes | No | No | No | Yes | No | No | No | No | No | No |
| Kivimäki, et al, 2010 [21] | Whitehall II usual measure model | No | No | No | Yes | Yes | Yes | Yes | Yes | Yes | No | No | No | Yes | No | No | No | No | No | No |
| Kshirsagar et al, 2010 [19] | ARIC/CHS model | Yes | Yes | No | Yes | Yes | No | Yes | Yes | Yes | No | Yes | No | Yes | No | Yes | Yes | No | No | No |
| Bozorgmanesh et al, 2011[23] | Iran model – Women | Yes | No | No | Yes | Yes | No | Yes | Yes | No | No | No | No | No | No | No | No | No | No | No |
| Bozorgmanesh et al, 2011[23] | Iran model – Men | Yes | No | No | Yes | Yes | No | No | No | No | No | No | No | No | No | No | No | No | No | No |
| Chien et al, 2011[24] | Taiwan model 1 (clinical model) | Yes | Yes | No | Yes | Yes | No | No | Yes | No | No | No | No | No | No | No | No | No | No | No |
| Chien et al, 2011[24] | Taiwan model 2 (biochemical model) | Yes | Yes | No | Yes | Yes | No | No | Yes | No | No | No | No | No | No | Yes | No | Yes | Yes | No |
| Lim et al, 2013 [25] | Korean risk model | Yes | Yes | No | Yes | Yes | No | Yes | Yes | Yes | No | No | No | No | No | No | No | No | No | No |
| Fava et al, 2013 [22] | Swedish risk model | Yes | Yes | No | No | No | No | Yes | Yes | Yes | Yes | Yes | No | No | Yes | Yes | No | No | No | Yes |

ARIC: Atherosclerosis Risk in Communities, Age*BP: interaction term of age and BP variables, Age*BP: interaction term of age and BP variables, Age*sex: interaction term of age and sex, BP: blood pressure, BMI: body mass index, CHS: Cardiovascular Health Study, CRP: C-reactive protein, DBP: diastolic blood pressure, HTN: hypertension, SBP: systolic blood pressure, WBC: white blood cell, WC: waist circumference, WHS: Women’s Health Study.
