# Supplementary material for: Risk Models to Predict Hypertension: A Systematic Review
Source: PLoS One. 2013 Jul 5;8(7):e67370. doi: 10.1371/journal.pone.0067370 (PMC3702558; doi:10.1371/journal.pone.0067370)
Supplement: Table S2 — Reporting and management of missing values in studies on the development of hypertension risk scores. (DOCX) [file pone.0067370.s003.docx]

**Table S2: Reporting and management of missing values in studies on the development of hypertension risk prediction models**

| **First author** | **Outcome** | |  | **Variables included in risk models** | |
| --- | --- | --- | --- | --- | --- |
|  | **Missing values** | **Management of missing values** |  | **Missing values** | **Handling of participants with missing values** |
| Pearson et al, 1990 [[1](#_ENREF_1)] | Not reported | Not applicable |  | Not reported | Not reported |
| Parikh et al,2008 [[2](#_ENREF_2)] | Not reported | Not applicable |  | Reported without specifying the variables affected | Exclusion of participants with missing values |
| Paynter et al, 2009 [[3](#_ENREF_3)] | Not reported | Not applicable |  | Reported without specifying the variables affected | Exclusion of participants with missing values |
| Kivimäki, et al, 2009 [[4](#_ENREF_4)] | Not reported | Not applicable |  | Reported without specifying the variables affected | Exclusion of participants with missing values |
| Kivimäki, et al, 2010 [[5](#_ENREF_5)] | Not reported | Not applicable |  | Reported without specifying the variables affected | Exclusion of participants with missing values |
| Kshirsagar et al, 2010 [[6](#_ENREF_6)] | Not reported | Not applicable |  | Reported without specifying the variables affected | Single imputation of missing values for family history of hypertension, exclusion of participants with missing values on other covariates |
| Bozorgmanesh et al, 2011 [[7](#_ENREF_7)] | Not reported | Not applicable |  | Not reported | Not reported |
| Chien et al, 2011 [[8](#_ENREF_8)] | Not reported | Not applicable |  | Not reported | Not reported |
| Lim et al, 2013 [[9](#_ENREF_9)] | Not reported | Not applicable |  | Reported without specifying the variables affected | Exclusion of participants with missing values |
| Fava et al, 2013 [[10](#_ENREF_10)] | Not reported | Not applicable |  | Reported only for genotype variables | Imputation of missing values for the genotype variables, exclusion of participants with missing values for other variables |

**References**

1. Pearson TA, LaCroix AZ, Mead LA, Liang KY (1990) The prediction of midlife coronary heart disease and hypertension in young adults: the Johns Hopkins multiple risk equations. Am J Prev Med 6: 23-28.

2. Parikh NI, Pencina MJ, Wang TJ, Benjamin EJ, Lanier KJ, et al. (2008) A risk score for predicting near-term incidence of hypertension: the Framingham Heart Study. Ann Intern Med 148: 102-110.

3. Paynter NP, Cook NR, Everett BM, Sesso HD, Buring JE, et al. (2009) Prediction of incident hypertension risk in women with currently normal blood pressure. Am J Med 122: 464-471.

4. Kivimaki M, Batty GD, Singh-Manoux A, Ferrie JE, Tabak AG, et al. (2009) Validating the Framingham Hypertension Risk Score: results from the Whitehall II study. Hypertension 54: 496-501.

5. Kivimaki M, Tabak AG, Batty GD, Ferrie JE, Nabi H, et al. (2010) Incremental predictive value of adding past blood pressure measurements to the Framingham hypertension risk equation: the Whitehall II Study. Hypertension 55: 1058-1062.

6. Kshirsagar AV, Chiu YL, Bomback AS, August PA, Viera AJ, et al. (2010) A hypertension risk score for middle-aged and older adults. J Clin Hypertens (Greenwich) 12: 800-808.

7. Bozorgmanesh M, Hadaegh F, Mehrabi Y, Azizi F (2011) A point-score system superior to blood pressure measures alone for predicting incident hypertension: Tehran Lipid and Glucose Study. J Hypertens 29: 1486-1493.

8. Chien KL, Hsu HC, Su TC, Chang WT, Sung FC, et al. (2011) Prediction models for the risk of new-onset hypertension in ethnic Chinese in Taiwan. J Hum Hypertens 25: 294-303.

9. Lim NK, Son KH, Lee KS, Park HY, Cho MC (2013) Predicting the risk of incident hypertension in a korean middle-aged population: korean genome and epidemiology study. J Clin Hypertens (Greenwich) 15: 344-349.

10. Fava C, Sjogren M, Montagnana M, Danese E, Almgren P, et al. (2013) Prediction of blood pressure changes over time and incidence of hypertension by a genetic risk score in Swedes. Hypertension 61: 319-326.
