# Supplementary material for: Risk Models to Predict Hypertension: A Systematic Review
Source: PLoS One. 2013 Jul 5;8(7):e67370. doi: 10.1371/journal.pone.0067370 (PMC3702558; doi:10.1371/journal.pone.0067370)
Supplement: Text S1 — Search terms for risk model development or validation studies. (DOCX) [file pone.0067370.s004.docx]

**Text S1: Search terms**

| **Database: PubMed** (Inception until April 2013) |
| --- |
| #1 hypertension AND “blood pressure”  #2 "pulmonary hypertension" OR "pulmonary arterial hypertension" OR "intracranial hypertension" OR "intraocular pressure" OR glaucoma OR "ocular hypertension" OR "intraocular hypertension" OR eclampsia OR "pre eclampsia" OR "pre-eclampsia" OR "gestational hypertension" OR "pregnancy-induced hypertension" OR "secondary hypertension" OR "renal hypertension" OR "renovascular hypertension" OR "portal hypertension" OR "intra-abdominal hypertension" OR "malignant hypertension" OR "white coat hypertension" OR "masked hypertension" OR "pseudohypoaldosteronism" OR "venous hypertension" OR "resistant hypertension" OR "hypercortisolism" OR "Cushing disease" OR "Cushing syndrome" OR "pheochromocytoma" OR "renal artery stenosis" OR "hyperaldosteronism" OR "Conn syndrome" OR "Conn disease"  #3 #1 NOT #2  #4 (validat* OR predict*[tiab] OR rule*) OR (predict* AND (outcome* OR risk* OR model*)) OR ((history OR variable* OR criteria OR scor* OR characteristic* OR finding* OR factor*) AND (predict* OR model* OR decision* OR identif* OR prognos*)) OR (decision* AND (model* OR clinical* OR logistic models)) OR (prognostic AND (history OR variable* OR criteria OR scor* OR characteristic* OR finding* OR factor* OR model*)) OR ("stratification" OR "ROC Curve"[MeSH] OR "discrimination" OR "discriminate" OR "c statistic" OR "area under the curve" OR "Calibration" OR "Indices" OR "algorithm" OR "Multivariable")  #5 “cohort” OR “observational” OR “prospective” OR “trial” OR “epidemiology”  #6 #3 AND #4 AND #5  #7 (Animals[MeSH] NOT Humans[MeSH])  #8 #6 NOT # 7 |
| **Database: EMBASE**  (Inception until April 2013) |
| #1 (hypertension AND “blood pressure”)  #2 "pulmonary hypertension" OR "intracranial hypertension" OR "intraocular hypertension" OR eclampsia OR "preeclampsia" OR "maternal hypertension" OR "renovascular hypertension" OR "portal hypertension" OR "intraabdominal hypertension" OR "malignant hypertension" OR "white coat hypertension" OR "masked hypertension" OR "resistant hypertension" OR "hypercortisolism" OR "Cushing disease" OR "Cushing syndrome" OR "pheochromocytoma" OR "kidney artery stenosis" OR " primary hyperaldosteronism"  #3 #1 NOT #2  #4 AND ((validat* OR predict* OR rule*) OR (predict* AND (outcome* OR risk* OR model*)) OR ((history OR variable* OR criteria OR scor* OR characteristic* OR finding* OR factor*) AND (predict* OR model* OR decision* OR identif* OR prognos*)) OR (decision* AND (model* OR clinical* OR logistic models)) OR (prognostic AND (history OR variable* OR criteria OR scor* OR characteristic* OR finding* OR factor* OR model*)) OR ("stratification" OR "ROC Curve" OR "discrimination" OR "discriminate" OR "c statistic" OR "area under the curve" OR "Calibration" OR "Indices" OR "algorithm" OR "Multivariable"))  #5“cohort analysis” OR “observational study” OR “prospective study” OR “clinical trial” OR “randomized controlled trial” OR “epidemiology”  #6 ([adult]/lim OR [aged]/lim) AND [humans]/lim AND [english]/lim  #7 #3 AND #4 AND #5 AND #6 |
