# Supplementary material for: Risk Models to Predict Hypertension: A Systematic Review
Source: PLoS One. 2013 Jul 5;8(7):e67370. doi: 10.1371/journal.pone.0067370 (PMC3702558; doi:10.1371/journal.pone.0067370)
Supplement: Text S2 — Search terms for risk model impact studies. (DOCX) [file pone.0067370.s005.docx]

**Text S2 – Search terms for impact studies**

Search terms used to identify all impact studies, which are combined with each specific risk scores acronym, or if not applicable the name of the cohort in which the score was developed or first author:

**Database: PubMed**(Inception until April 2013)

(Effectiveness [tiab] OR Comparing [tiab] OR Compared [tiab] OR Evaluate [tiab]) AND (Algorithm [tiab] OR Strategy [tiab] OR Managed [tiab] OR Management [tiab] OR Decision [tiab]) AND (hypertension AND "blood pressure") NOT (Animals [MeSH] NOT Humans[MeSH]).

**Database: EMBASE**(Inception until April 2013)

(Effectiveness:ti,ab OR Comparing:ti,abOR Compared:ti,ab OR Evaluate:ti,ab) AND (Algorithm:ti,ab OR Strategy:ti,ab OR Managed:ti,ab OR Management:ti,ab OR Decision:ti,ab) AND ("hypertension" AND "blood pressure ") AND [humans]/lim
